# *Elephantorrhiza elephantina*: Traditional Uses, Phytochemistry, and Pharmacology of an Important Medicinal Plant Species in Southern Africa

**DOI:** 10.1155/2017/6403905

**Published:** 2017-05-14

**Authors:** Alfred Maroyi

**Affiliations:** Department of Botany, University of Fort Hare, Private Bag X1314, Alice 5700, South Africa

## Abstract

*Elephantorrhiza elephantina* is used in southern Africa as traditional remedy for a wide range of human diseases and ailments including dermatological diseases, gastrointestinal system disorders, sexual dysfunction, sexually transmitted infections, and wounds. The rhizome decoction of* E. elephantina* is widely used by small-scale farmers in Botswana and South Africa as ethnoveterinary medicine for cattle, goats, horses, pigs, poultry, and sheep. Several classes of phytochemical compounds including anthocyanidins, anthraquinones, esters, fatty acids, phenolic compounds, flavonoids, glycosides, polysterols, saponins, sugars, tannins, and triterpenoids have been isolated from* E. elephantina*. Scientific studies on* E. elephantina* indicate that it has a wide range of biological activities including anthelmintic, antibacterial, antifungal, anti-inflammatory and antinociceptive, antiplasmodial, antioxidant, antibabesial, and antirickettsial activities.* Elephantorrhiza elephantina* is a valuable source of traditional medicine in southern Africa that it is worth additional research attention because of its wide ethnomedicinal applications and promising biological activities. However, the current health-related information on* E. elephantina* is not sufficiently explored as diverse studies on its chemical and pharmacological activities are required to understand its mechanism of action and to characterize the metabolites responsible for these activities.

## 1. Introduction

*Elephantorrhiza elephantina* (Burch.) Skeels is a member of a small and purely African genus represented by nine species on the continent [1].* Elephantorrhiza elephantina* is the type species of the genus, where the generic name “Elephantorrhiza” means “elephant root” and is based, most descriptively, on the large underground stem common to most members of the genus [2]. Four species in this genus, namely,* E. burkei* Benth.,* E. elephantina*,* E. goetzei* (Harms) Harms, and* E. suffruticosa* Schinz, are highly regarded as medicinal plants in southern Africa [3–5].* Elephantorrhiza elephantina* is an important plant resource in southern Africa, where it provides food and medicine for the indigenous people and the bark of its tuberous rhizome is a popular source of tanning and dyeing materials [6]. The underground rhizomes, often referred to as roots, are one of the primary herbal medicines in southern Africa. Due to its popularity as herbal medicine,* E. elephantina* is sold as herbal medicine in the herbal medicine (muthi) markets in Botswana and Zimbabwe [7], the Eastern Cape province of South Africa [8, 9], Gauteng province [10], Limpopo province [11], and the Northern Cape province [12]. According to Dold and Cocks [8], the average price of* E. elephantina* per kg in the Eastern Cape province is R30.80 (US$3.60) and 108.80 kg is the mean quantity sold per trader per year. Due to high demand of the species as herbal medicine and also because harvesters mainly target the rhizomes,* E. elephantina* is recorded in the Red Data List of Lesotho as data deficient by Talukdar [13] based on the utilization of the species as herbal medicine for stopping bleeding, treating syphilis and intestinal disorders. van Wyk [14] listed* E. elephantina* as a plant species of high importance with its roots having potential in the formulation of commercial herbal medicines as antioxidant, skin ailments, diarrhoea, perforated ulcers, prostrate hypertrophy, and male pattern baldness in South Africa. In South Africa,* E. elephantina* is used as a traditional remedy for a wide range of ailments, including diarrhoea and dysentery, stomach disorders, skin diseases and acne, haemorrhoids, and perforated peptic ulcers and as emetics [15]. Rhizomes or bark of* E. elephantina* is crushed with some water added; the resulting paste is applied to hides to tan and dye them a reddish colour [16]. The young shoots of* E. elephantina* are eaten by livestock and its seeds have a sweetish taste followed by a burning sensation and are often roasted in southern Africa as a coffee substitute [16].

With the increasing realization worldwide that traditional medicines based on indigenous medical systems are potential sources of natural products that can be developed into pharmaceutical drugs and health products [14], substantial efforts have been made to investigate ethnomedicinal uses, chemical constituents, and biological activities of* E. elephantina* during the last three decades. Unfortunately, no comprehensive review on this important plant species in southern Africa has been published, documenting the species' biology, traditional uses, phytochemistry, and pharmacological properties. Therefore, in this study, the advances in traditional utilization, botany, phytochemistry, pharmacology, and safety aspects of* E. elephantina* are systematically reviewed. In addition to this, the perspectives for the future research on* E. elephantina* are also discussed in the hope that the article will provide a better understanding of the plant species.

## 2. Methodology of the Review

The literature search was performed from March 2016 to January 2017 using electronic search engines such as Google and Google Scholar and publishing sites such as Elsevier, Science Direct,* BioMed Central* (BMC), and PubMed. The databases and literature sources were chosen based on the topics covered (i.e., biological activities, ethnobotany, ethnomedicinal uses, ethnopharmacology, pharmacology, phytochemistry, and therapeutic value) and geographical coverage (i.e., southern Africa). The following keywords were used to search literature sources:* Acacia elephantina*,* Elephantorrhiza burchellii* and* Elephantorrhiza elephantina*, dwarf elephant's root, eland's bean, eland's wattle, and elephant's foot. Other literature sources included papers published in international journals, reports from international, regional, and national organizations, conference papers, books, theses, websites, and other grey literature. References were also identified by searching the library collections of the National Herbarium and Botanic Gardens (SRGH), Harare, Zimbabwe, and the University of Fort Hare, South Africa.

## 3. Species Description

*Elephantorrhiza elephantina* has been recorded in southern Africa, that is, Namibia, Botswana, Zimbabwe, Mozambique, Swaziland, Lesotho, and South Africa.* Elephantorrhiza elephantina* is usually widespread, often gregarious and forming huge patches in hot and dry areas in grasslands and open scrub [6]. Synonyms of* E. elephantina* are* Acacia elephantina* Burch. and* Elephantorrhiza burchellii* Benth.* Elephantorrhiza elephantina* is a perennial suffrutex or low shrub, producing annual stems up to 90 cm tall at ground level, from the woody end of an elongate, with often thickened rhizome up to 8 m long [16]. Its leaves are alternate, bipinnately compound, almost glabrous with a petiole up to 8 cm long [17]. The leaves consist of 2–4 pairs of pinnae in lower leaves and 7–17 pairs in upper ones, where the axis is up to 10 cm long. The leaflets are up to 55 pairs per pinna, linear to oblong in shape, 4–15 mm long and 0.50–2.50 mm wide with an asymmetric base, apex acute, and usually mucronate [17].* Elephantorrhiza elephantina* inflorescence is an axillary raceme, usually confined to the lower part of the stem usually solitary or clustered. The flowers are bisexual with red-brown glands at the base and free petals which are slightly connate at the base. The petals are linear-oblong, 2–4 mm long and about 1 mm wide, and yellow-white in colour [16]. The stamens are 10 which are free, with filaments up to 6.50 mm long [17]. The fruit is a compressed-oblong, straight or slightly curved pod 5–21 cm long and 3–6 cm wide, red-brown in colour, prominently transversely veined, and often swollen over the seeds [16].

## 4. Vernacular Names of* Elephantorrhiza elephantina*

*Elephantorrhiza elephantina* is known by several vernacular names in its geographical areas of occurrence (Table 1). Literature survey showed no fewer than 41 common or vernacular names for* E. elephantina* in the seven countries where it is indigenous (Table 1). Local people rarely name plant species that they do not use [18]. This list of common or vernacular names implies that local people in southern Africa have an active interest in* E. elephantina*. South Africa has the highest number of common or vernacular names (21 in total) followed by Botswana (seven), Namibia (five), and Zimbabwe with four names and the rest of the countries have either one or two names (Table 1). A vernacular name often describes some characteristic feature of the plant species or the plant parts, for example, “eland's bean” (an eland is an indigenous gazelle species); “*elandsboontjie*”; “eland's wattle”; “elephant's foot”; “elephant-root”; or “dwarf elephant's root” (Table 1).* Elephantorrhiza elephantina* is commonly referred to as “*elandsboontjie*” in Afrikaans in South Africa and “*eland's bean*” and “*eland's wattle*” in English in Namibia and South Africa because elands feed on the species foliage and pods [19]. The other English common names, “elephant's foot” and “elephant-root,” are in reference to large and long rhizomes or roots of the species measuring up to 8 m long [2]. The common name “dwarf elephant's root” is in reference to the height of* E. elephantina*, which rarely exceeds one metre in height [17, 20] in comparison to a closely related species* E. goetzei* also known as “elephant's root” but averaging seven metres in height [5, 6].

## 5. Ethnomedicinal Uses of* Elephantorrhiza elephantina*

The rhizome, roots, leaves, and stems of* E. elephantina* are reported to possess diverse medicinal properties and are used to treat or manage various human and animal ailments and diseases throughout its distributional range in southern Africa (Table 2). A total of 42 and 14 human and animal ailments and diseases, respectively, are treated by herbal medicines prepared from* E. elephantina* (Table 2). These reports are from all the countries where* E. elephantina* is indigenous. The country with the highest ethnomedicinal uses is South Africa (45) based on 25 literature records, followed by Lesotho with ten uses and two literature records, Botswana with nine uses and four literature records, Mozambique and Zimbabwe with five uses and two literature records each, and Namibia and Swaziland with a single use and literature record each.* Elephantorrhiza elephantina* is mainly used to treat disorders of the gastrointestinal tract (21 citations in six countries), followed by veterinary medicine (14 citations in two countries), skin diseases (six citations from South Africa only), pain (five citations in five countries), and infertility and impotence (five citations in four countries). These records show high degree of consensus for the major diseases and ailments (Table 2) and imply high cross-cultural agreement among ethnomedicinal uses of* E. elephantina* throughout its distributional range.

The rhizome or root decoction of* E. elephantina* is used to relieve abdominal pains in Lesotho and Zimbabwe [3, 24] and chest pains in South Africa [42] and applied to open wounds to stop bleeding [39]. In South Africa, roots and rhizomes of* E. elephantina* are boiled in water for external use to treat acne and other skin diseases [36–38] while roots and rhizomes of* E. elephantina* in combination with* Pentanisia prunelloides* (Klotzsch & Eckl. & Zeyh.) Walp. are used to treat eczema [36, 37]. Roots or rhizome decoction of* E. elephantina* is taken orally as remedy for various ailments and diseases including anemia in Mozambique [25], blood pressure, clearing air canal, erectile dysfunction, haemorrhoids, itching, kidney failure, intestinal disorders, menstrual disorders, peptic ulcers, rheumatic conditions, shingles, sores, syphilis, and tonsillitis in South Africa [4, 12, 15, 31, 38, 44, 47, 48, 50]. In Botswana, rhizome or root powder of* E. elephantina* is used to wipe the anus of children with bloody diarrhoea, to clean the womb after abortion, as remedy for early menstruating children, earache, erectile dysfunction, and sexually transmitted infections [21, 40, 41]. In Lesotho, rhizome decoction of* E. elephantina* is used to cleanse blood, as remedy for breast cancer, herpes, infertility, intestinal disorders, stomach problems, syphilis, and tuberculosis [24, 39]. Leaf, rhizome, and root decoction of* E. elephantina* are used as remedy for diarrhoea and dysentery in Mozambique [45] and South Africa [15, 28, 29, 32, 38, 44, 46]. In some cases in South Africa, the rhizome of* E. elephantina* is mixed with* Acokanthera oblongifolia* Benth. & Hook.f. ex B.D. Jacks root as remedy for diarrhoea and stomach ailments in South Africa [44]. In Mozambique, root decoction of* E. elephantina* is taken orally as a pain killer [25] and for sexually transmitted infections [45].

*Elephantorrhiza elephantina* root decoction is taken orally as emetics for fever in Mozambique [25] and South Africa [42]. The rhizome of* E. elephantina* is mixed with roots of* Pentanisia prunelloides* and taken orally as remedy for fever and stomach ailments in Zimbabwe [43].* Elephantorrhiza elephantina* is an ingredient of a herbal mixture known as “Sejeso” (Ingwe® brand) made up of* Alepidea amatymbica* Eckl. & Zeyh.,* Hypoxis obtusa* Burch. ex Ker Gawl.,* Pentanisia prunelloides*, deionized water, and potassium sorbate as preservative used as remedy for constipation, heartburn, indigestion, loss of appetite, stomach ailments, and vomiting [43]. According to Semenya et al. [49], the rhizome of* E. elephantina* is mixed with roots of* Boscia albitrunca* (Burch.) Gilg & Gilg-Ben.,* Peltophorum africanum* Sond., and* Plectranthus ciliatus* E. Mey. as remedy for HIV/AIDS opportunistic infections. Research by de Wet et al. [51] revealed that* E. elephantina* root decoction is taken orally in combination with* Cladostemon kirkii* (Oliv.) Pax & Gilg (roots),* Drimia delagoensis* (Baker) Jessop (bulb),* Sarcophyte sanguinea* Sparm. ssp.* piriei* (Hutch.) B. Hansen (bark), and* Ranunculus multifidus* Forssk. (whole plant) as remedy for shingles. Research by de Wet et al. [51] also revealed that* E. elephantina* root decoction is taken orally in combination with* Cladostemon kirkii* (root),* Drimia delagoensis* (bulb),* Ficus sur* Forssk. (bark),* Ranunculus multifidus* (whole plant),* Sarcophyte sanguinea *ssp.* piriei,* and* Senecio serratuloides* DC. (leaves) as remedy for sores.

Rhizome decoction of* E. elephantina* is widely used by small-scale farmers in Botswana and South Africa as ethnoveterinary medicine for poultry and retained placenta in cattle and as ethnoveterinary medicine for other animals such as goats, horses, pigs, and sheep and for diseases such as black quarter, appetite stimulant, coughing, diarrhoea, gastrointestinal parasites, gall sickness, heartwater, mange, pneumonia, and ectoparasites [22, 23, 27, 30, 33, 48, 52, 53]. The young shoots of* E. elephantina* are eaten by livestock and wild animals in southern Africa [6]. In Namibia, the pods of* E. elephantina* are eaten by both people and animals [26].

## 6. Phytochemistry

**Figure 1:**
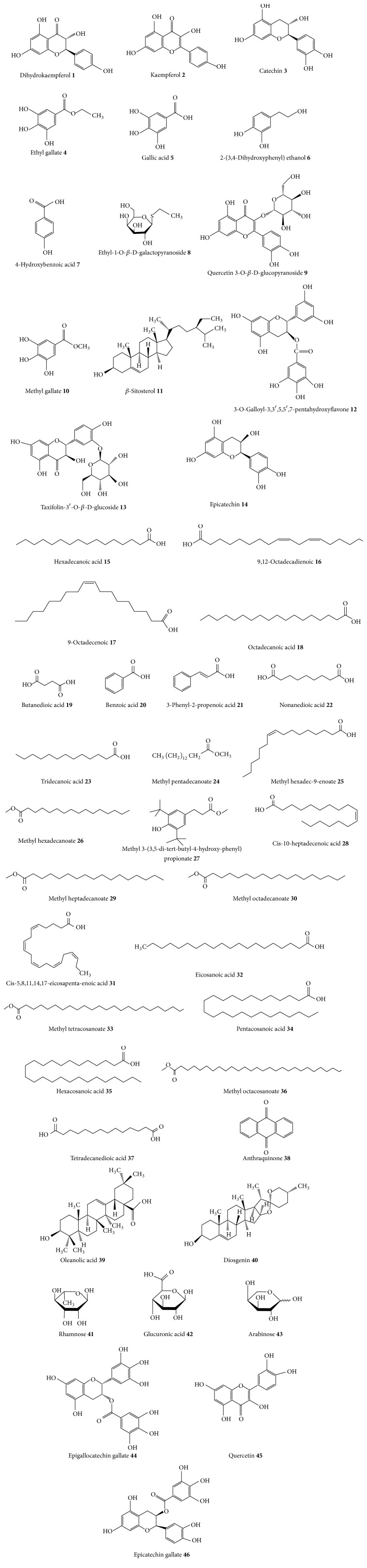
Chemical structures of major compounds isolated from rhizomes or roots of* Elephantorrhiza elephantina*.

Multiple classes of phytochemicals including anthocyanidins, anthraquinones, esters, fatty acids, phenolic compounds, flavonoids, glycosides, polysterols, saponins, sugars, tannins, and triterpenoids have been isolated from rhizome extracts of* E. elephantina* [41, 54–57]. Considerable pharmacological potential of* E. elephantina* has been documented through detection, isolation and purification of its natural products via advances in spectrometric techniques such as attenuated total reflection (ATR), Fourier transform infrared (FTIR) spectroscopy, liquid chromatography electron spray ionization mass spectroscopy (LC-ESI-MS), gas chromatography-mass spectrometry (GC-MS), and nuclear magnetic resonance (NMR) for structural elucidation of new and complex compounds (Table 3). Advanced research through ATR, LC-ESI-MS, FTIR, GC-MS, and NMR spectroscopy enabled researchers to have a better understanding of the correlations between molecular conformation and biological activities of the natural compounds of* E. elephantina* and its importance as herbal medicine. The compounds isolated from* E. elephantina* are documented and listed in Table 3 and their structures are displayed in Figure 1. Aaku et al. [41] isolated the following compounds from n-butanol rhizome extracts of* E. elephantina*: dihydrokaempferol** 1**, kaempferol** 2**, (−)-catechin** 3**, ethyl gallate** 4**, gallic acid** 5**, 2-(3,4-dihydroxyphenyl) ethanol** 6**, 4-hydroxybenzoic acid** 7**, ethyl-1-O-*β*-D-galactopyranoside** 8**, and quercetin 3-O-*β*-D-glucopyranoside** 9**. Phytochemical study of* E. elephantina* rhizomes by Mthembu [57] showed the presence of several phenolic compounds including catechin** 3**, gallic acid** 5**, quercetin 3-O-*β*-D-glucopyranoside** 9**, methyl gallate** 10**, *β*-sitosterol** 11**, 3-O-galloyl-3,3′,5,5′,7-pentahydroxyflavone** 12**, taxifolin-3′-O-*β*-D-glucoside** 13,** and epicatechin** 14**. Recently, Msimanga et al. [55] isolated the following compounds from hexane root extracts of* E. elephantina*: hexadecanoic** 15**, 9,12-octadecadienoic** 16**, 9-octadecenoic** 17**, octadecanoic acid** 18**, butanedioic acid** 19**, benzoic acid** 20**, 3-phenyl-2-propenoic acid** 21**, nonanedioic acid** 22**, tridecanoic acid** 23**, methyl pentadecanoate** 24**, methyl hexadec-9-enoate** 25**, methyl hexadecanoate** 26**, methyl 3-(3,5-di-tert-butyl-4-hydroxy-phenyl)propionate** 27**, cis-10-Heptadecenoic acid** 28**, methyl heptadecanoate** 29**, methyl octadecanoate** 30**, cis-5,8,11,14,17-eicosapenta-enoic acid** 31**, eicosanoic acid** 32**, methyl tetracosanoate** 33**, pentacosanoic acid** 34**, hexacosanoic acid** 35**, methyl octacosanoate** 36,** and tetradecanedioic acid** 37**. The phytochemical studies of the rhizome extracts of* E. elephantina* carried out by Mpofu et al. [54] showed the presence of anthraquinone** 38**, triterpenoids oleanolic acid** 39**, diosgenin** 40**, rhamnose** 41**, glucuronic acid** 42,** and arabinose** 43**. In another phytochemical evaluation of* E. elephantina* rhizome extracts, Mpofu et al. [56] isolated kaempferol** 2**, epicatechin** 14**, glucuronic acid** 42**, arabinose** 43**, epigallocatechin gallate** 44**, quercetin** 45,** and epicatechin gallate** 46**. The major phytochemical compounds isolated from* E. elephantina* are mainly fatty acids (39.13% of all known compounds isolated from the species), followed by phenolic compounds (26.09%) and esters (13.04%) and the contribution of the rest of the compounds is less than 10% each; see Table 3.

## 7. Pharmacological Activities

A number of pharmacological activities of* E. elephantina* have been reported in literature corroborating some of the ethnomedicinal uses listed in Table 2. Some of the pharmacological activities of* E. elephantina* listed in literature include anthelmintic [58–60], antibacterial [21, 28, 41, 43, 50, 61], antifungal [21, 41, 50, 61], anti-inflammatory and antinociceptive [62], antiplasmodial [63], antioxidant [54], and antibabesial and antirickettsial [64, 65] activities.

### 7.1. Anthelmintic Activity

Maphosa et al. [58] evaluated in vitro anthelmintic activities of crude aqueous extracts of* E. elephantina* roots on the eggs and larvae of the nematode parasite* Haemonchus contortus* using Valbazen® (11.36% albendazole) at 10 mg/kg and 0.5 mL/kg distilled water as positive and negative controls, respectively.* Elephantorrhiza elephantina* had 100% egg hatch inhibition at a concentration as low as 2.5 mg/mL. At the lowest concentration of 0.63 mg/mL tested,* E. elephantina* inhibited egg hatching by >96% and this was comparable to albendazole at the same concentration [58].* Elephantorrhiza elephantina* had complete inhibition of larval development at a concentration of 1.25 mg/mL [58]. This study by Maphosa et al. [58] demonstrated that inhibition of egg hatching and larval development increased significantly with increasing concentration of* E. elephantina* root extract. In another study, Maphosa and Masika [59] evaluated efficacy of* E. elephantina* aqueous root extracts in naturally mixed infections of gastrointestinal worms and Coccidia species in goats that had not been dosed for a period of two months, using Valbazen (11.36% albendazole) at 10 mg/kg and 0.5 mL/kg distilled water as positive and negative controls, respectively. In this study,* E. elephantina* caused reduction of* Trichuris* eggs on days 3 and 6 at 250 mg/kg dose. This study also revealed efficacy of* E. elephantina* against strongyle and* Eimeria* spp. at 500 mg/kg. The reduction in faecal egg counts in dosed extracts with* E. elephantina* against mixed gastrointestinal parasite infections shows that this species possess anthelmintic properties and there is credence in its ethnoveterinary use against gastrointestinal parasites in goats. In another study, Maphosa and Masika [60] evaluated anthelmintic activity of aqueous, hexane, and ethyl root extract of* E. elephantina* against adult* Haemonchus contortus* using a bioactivity-guided assay with albendazole and distilled water as positive and negative controls, respectively. The aqueous and ethyl acetate fractions showed high motility inhibition at concentrations of 2.50 mg/mL and above after 6-hour exposure, while the hexane fraction showed motility inhibition at concentrations of 5 mg/mL and above. After 30-hour exposure, all the fractions, that is, aqueous, hexane, and ethyl acetate fractions, and albendazole (commercial drug) showed inhibition of motility and the mortality indexes were not significantly different from each other [60]. All the anthelmintic evaluations carried out so far [58–60] confirmed the anthelmintic activities of the root of* E. elephantina*, a plant species widely used as anthelmintic remedy by small-scale farmers in South Africa.

### 7.2. Antibacterial Activity

Aaku et al. [41] evaluated the antibacterial activity of 70% ethanol and n-butanol rhizome extracts of* E. elephantina* using the thin-layer chromatography (TLC) bioautography technique with chloramphenicol and miconazole as positive and negative controls, respectively. Both extracts showed activity against* Bacillus subtilis*,* Escherichia coli*,* Pseudomonas aeruginosa,* and* Staphylococcus aureus* at loadings lower than 15 *μ*g. Among the purified compounds, only ethyl gallate** 4** and gallic acid** 5** showed activity against* Bacillus subtilis* and* Staphylococcus aureus* at loadings lower than 50 *μ*g. Similar results were obtained by Cueva et al. [66] who assessed the influence of pure phenolic compounds such as catechin** 3**, ethyl gallate** 4**, gallic acid** 5,** and epicatechin** 14** on the inhibition of the growth of potential respiratory pathogens. These authors found that nonflavonoid compounds such as ethyl gallate** 4** and gallic acid** 5** were more active than flavonoids such as catechin** 3** and epicatechin** 14**.

Mathabe et al. [28] evaluated the antibacterial activities of aqueous, acetone, ethanol, and methanol root extracts of* E. elephantina* against bacteria that cause gastrointestinal infections, namely,* Staphylococcus aureus*,* Vibrio cholerae*,* Shigella dysentery*,* Shigella sonnei*,* Shigella flexneri*, and* Shigella boydii*, and the minimum inhibitory concentration (MIC) of active extracts was determined by the microplate dilution assay. Mathabe et al. [28] used ten microliters of dimethyl sulfoxide (DMSO) per well as negative control while discs (5 mm in diameter) of nalidixic acid (30 mg), erythromycin (15 mg), and cotrimoxazole (25 mg) were used as positive controls. MIC activities against the pathogens ranged between 0.08 and 0.63 mg/mL, and the highest inhibition was exhibited against* Shigella flexneri* with MIC values ranging from 0.08 to 0.16 mg/mL [28], and these findings somehow confirm the species' antibacterial potential and its usefulness in the treatment and management of gastrointestinal infections. Mukanganyama et al. [21] evaluated antibacterial activities of ethanol root extracts of* E. elephantina* against* Bacillus cereus*,* Bacillus subtilis*,* Escherichia coli*,* Pseudomonas aeruginosa,* and* Staphylococcus aureus* using the agar diffusion assay. The species exhibited antibacterial properties against all microorganisms tested and the authors assessed the minimal inhibitory concentrations (MICs) against* Mycobacterium aurum*, where* E. elephantina* showed some activity with MIC value of 1.25 mg/mL [21].

Mabona et al. [61] evaluated antibacterial activities of aqueous and dichlomethane/methanol (1 : 1) leaf, root, and rhizome extracts of* E. elephantina* using the micro-titre plate dilution technique against dermatologically relevant pathogens such as* Brevibacillus agri*,* Propionibacterium acnes*,* Pseudomonas aeruginosa*,* Staphylococcus aureus* and* Staphylococcus epidermidis* with ciprofloxacin as positive control and acetone and dimethyl sulfoxide (DMSO) as negative controls. Mabona et al. [61] found varied antibacterial activities of the aqueous and dichlomethane/methanol (1 : 1) leaf, root and rhizome extracts with minimum inhibition concentration (MIC) ranging from 0.05 to >16.00 mg/mL. Antibacterial activities were displayed by dichlomethane/methanol leaf, root and rhizome extracts against* Propionibacterium acnes* (MIC values ranging from 0.05 to 1.00 mg/mL),* Staphylococcus aureus* (0.50 mg/mL) and* Staphylococcus epidermis* (0.38 to 1.00 mg/mL) as well as aqueous and dichlomethane/methanol root and rhizome extracts against* Brevibacillus agri* with MIC value of 0.50 mg/mL. The leaf, root and rhizome extracts of* E. elephantina* are reported to be traditionally used to treat acne vulgaris and pimples and such usage was corroborated by noteworthy activity against* Propionibacterium acnes* with MIC values between 0.05 and 2.0 mg/mL [61].* Propionibacterium acnes* is an important skin pathogen responsible for the chronic inflammatory disease of the sebaceous glands and hair follicles of the skin [61]. The aqueous root extracts of* Pentanisia prunelloides* combined (1 : 1) with* E. elephantina* displayed synergistic interactions with sum of the fractional inhibitory concentration (ΣFIC) values ranging from 0.31 to 0.38 mg/mL against* Staphylococcus aureus* and* Staphylococcus epidermidis*. The synergistic interactions noted for* Pentanisia prunelloides* and* E. elephantina* by Mabona et al. [61] validate their antibacterial effects as these two species are often used in combination as herbal medicines for treating microbial infections in southern Africa. Similarly, Nciki et al. [50] evaluated antibacterial activities of aqueous and dichlomethane/methanol (1 : 1) root extract of* E. elephantina* using the micro-titre plate dilution technique against dermatologically relevant pathogens such as* Brevibacillus agri*,* Escherichia coli*,* Propionibacterium acnes*,* Pseudomonas aeruginosa*,* Staphylococcus aureus* and* Staphylococcus epidermidis* with ciprofloxacin as positive control. Best antimicrobial results were demonstrated by dichlomethane/methanol extracts against* Escherichia coli* with MIC value of 130 *μ*g/mL,* Brevibacillus agri* (MIC value of 250 *μ*g/mL),* Propionibacterium acnes* (MIC value of 250 *μ*g/mL) and* Pseudomonas aeruginosa* with MIC value of 250 *μ*g/mL [50]. Nciki et al. [50] and Mabona et al. [61] obtained similar results in terms of overall antibacterial activities displayed against* Brevibacillus agri*,* Propionibacterium acnes* and* Pseudomonas aeruginosa* although Nciki et al. [50] also assessed the antibacterial activities of* E. elephantina* against* Escherichia coli*. Nciki et al. [50] assessed antibacterial activities of aqueous and dichlomethane/methanol (1 : 1) root extracts of* E. elephantina* while Mabona et al. [61] assessed antibacterial activities of other plant parts which included leaves and rhizomes. Therefore, the results obtained by both Nciki et al. [50] and Mabona et al. [61] provide a scientific rational for the traditional use of* E. elephantina* as herbal medicine against several skin infections in South Africa such as acne [15, 36–38], eczema [36, 37], itching [12], sores [50, 51] and sunburn [15, 38].

Mpofu et al. [43] evaluated antibacterial activity of the methanol and aqueous rhizome extracts of* E. elephantina* using the micro-titre plate dilution technique against* Bacillus cereus*,* Enterococcus faecalis* and* Escherichia coli* with ciprofloxacin as positive control and distilled water and dimethyl sulfoxide (DMSO) as negative controls. The minimum inhibitory concentration (MIC) values for the aqueous (0.50–2.00 mg/mL) and methanol (0.20–4.00 mg/mL) extracts independently demonstrated varied efficacies depending on the pathogen of study. Mpofu et al. [43] also evaluated the antibacterial activity of* E. elephantina* with* Pentanisia prunelloides* combined in 1 : 1 ratios, displaying synergistic interactions with sum of the fractional inhibitory concentration (ΣFIC) values ranging from 0.19 to 1.00 mg/mL for aqueous extracts and 0.60 to 0.80 mg/mL for methanol extracts against* Bacillus cereus*,* Enterococcus faecalis* and* Escherichia coli*. The antibacterial activity of* E. elephantina* in combination with* Pentanisia prunelloides* were determined as a validation of their combined use in southern African traditional medicine. Mpofu et al. [43] also evaluated the antibacterial activity of epicatechin** 14** and hexadecanoic acid** 15** isolated from* E. elephantina* rhizomes using the microtitre plate dilution technique against* Bacillus cereus*,* Enterococcus faecalis* and* Escherichia coli* with ciprofloxacin as positive control and distilled water and dimethyl sulfoxide (DMSO) as negative controls. The efficacy for the two compounds measured via MIC values ranged between 0.13 and 0.63 mg/mL, while synergistic interactions were noted against* Escherichia coli* and* Enterococcus faecalis* with (ΣFIC) values of 0.09 mg/mL and 0.50 mg/mL, respectively [43]. Therefore, the two compounds epicatechin** 14** and hexadecanoic acid** 15** showed synergistically enhanced activity especially against* Escherichia coli* and* Enterococcus faecalis*. Furthermore, previous studies have shown that hexadecanoic acid** 15** is active against various bacterial strains [67] including* Escherichia coli* [68] and epicatechin** 14** is also active against* Escherichia coli* and can play an important role in developing pharmaceutical drugs against urinary tract infections [69]. Epicatechin** 14** has also been implicated for antibacterial activity against* Escherichia coli*,* Bacillus cereus*,* Staphylococcus aureus,* and* Shigella flexneri* at minimum inhibition concentration (MIC) values ranging from 12.50 to 100 mg/mL [70, 71]. The antibacterial potency of this compound isolated from* E. elephantina* is noteworthy as the species is administered as a remedy by traditional healers in Botswana [40, 41], Mozambique [45], South Africa [15, 28, 29, 32, 38, 44, 46], and Swaziland [34]. These results support the traditional use of* E. elephantina* in treating bacterial infections such as diarrhoea and sexually transmitted infections.

### 7.3. Antifungal Activity

Aaku et al. [41] evaluated the antifungal activity of 70% ethanol and n-butanol rhizome extracts of* E. elephantina* using the TLC bioautography technique with chloramphenicol and miconazole as positive and negative controls, respectively. Both extracts showed activity against* Candida mycoderma* at loadings lower than 15 *μ*g. These results support the traditional use of* E. elephantina* in treating fungal infections associated with gastrointestinal tract infections. Mukanganyama et al. [21] evaluated antifungal activities of root ethanol extracts of* E. elephantina* against* Candida albicans* and* Candida mycoderma* using the agar diffusion assay. The species exhibited antifungal properties against both microorganisms tested and the authors assessed the minimal inhibitory concentrations (MICs) against* Candida albicans* and* E. elephantina* showed some activity with MIC value of 1.25 mg/mL [21]. Mabona et al. [61] evaluated antifungal activities of aqueous and dichlomethane/methanol (1 : 1) extracts of* E. elephantina* using the microtitre plate dilution technique against dermatologically relevant pathogens such as* Candida albicans*,* Microsporum canis,* and* Trichophyton mentagrophytes* with amphotericin B as positive control and acetone and dimethyl sulfoxide (DMSO) as negative controls. Mabona et al. [61] found varied antifungal activities of the aqueous and dichlomethane/methanol (1 : 1) leaf, root, and rhizome extracts with minimum inhibition concentration (MIC) 0.05 to >16.00 mg/mL. Noteworthy antifungal activities were displayed by dichlomethane/methanol leaf, root, and rhizome extracts against* Microsporum canis* (0.50 mg/mL),* Candida albicans* (1.00 mg/mL), and* Trichophyton mentagrophytes* (1.00 mg/mL). The aqueous root extracts of* Pentanisia prunelloides* combined (1 : 1) with* E. elephantina* displayed synergistic interactions with sum of the fractional inhibitory concentration (ΣFIC) values ranging from 0.31 to 0.38 mg/mL against* Candida albicans*. The synergistic interactions noted for* Pentanisia prunelloides* and* E. elephantina* by Mabona et al. [61] validate their antifungal effects as these two species are often used in combination as herbal medicines to treat skin infections. Similarly, Nciki et al. [50] evaluated antifungal activities of aqueous and dichlomethane/methanol (1 : 1) root extract of* E. elephantina* using the microtitre plate dilution technique against dermatologically relevant pathogens such as* Candida albicans*,* Microsporum canis,* and* Trichophyton mentagrophytes* with amphotericin B as positive control. Best antifungal results were demonstrated by dichlomethane/methanol extracts against* Candida albicans* with MIC value of 130 *μ*g/mL,* Microsporum canis* (MIC value of 250 *μ*g/mL), and* Trichophyton mentagrophytes* with MIC value of 250 *μ*g/mL [50]. It is important to note that Nciki et al. [50] assessed antifungal activities of root extracts only while Mabona et al. [61] evaluated antifungal roots, leaves, and rhizomes of* E. elephantina*. There are also differences in terms of best antifungal results documented in these two studies. According to Mabona et al. [61] the best antifungal activities were demonstrated by dichlomethane/methanol leaf, root, and rhizome extracts against* Microsporum canis* with MIC value of 0.50 mg/mL while best antifungal results obtained by Nciki et al. [50] were demonstrated by dichlomethane/methanol extracts against* Candida albicans* with MIC value of 130 *μ*g/mL. Overall, results obtained by Nciki et al. [50] and Mabona et al. [61] provide a scientific basis for the traditional use of* E. elephantina* as herbal medicine against several skin infections in South Africa such as acne [15, 36–38], eczema [36, 37], itching [12], sores [50, 51], and sunburn [15, 38].

### 7.4. Anti-Inflammatory and Antinociceptive Activities

Maphosa et al. [62] evaluated anti-inflammatory and antinociceptive activities of root extract of* E. elephantina* using Wistar rats. The authors evaluated anti-inflammatory activities using carrageenan and histamine-induced rat paw oedema while antinociceptive activity was evaluated by acetic acid-induced writhing test and formalin test. The aqueous extract of* E. elephantina* reduced the formation of oedema induced by carrageenan and histamine and caused reduction in writhings in the acetic acid test and licking time in the formalin test [62]. According to Maphosa et al. [62], the root extract of* E. elephantina* reduced oedema and pain even better than the control, indomethacin, a potent inhibitor of prostaglandins (PG) synthesis, showing that the plant species has strong anti-inflammatory and antinociceptive activities. The anti-inflammatory activity displayed by root extract of* E. elephantina* could be due to anthraquinone** 38**, as previous research by Mishchenko et al. [72] showed that cell culture composed of anthraquinone** 38** isolated from* Rubia cordifolia* L. exhibited anti-inflammatory activity, which is manifested by an antiexudative effect and antiproliferative action during the rapid development of a model edema. These results support the traditional use of the species in various inflammatory ailments and diseases ranging from microbial infections to sores and wounds that result in cell injury and pain.

### 7.5. Antiplasmodial Activity

Clarkson et al. [63] evaluated aqueous, dichloromethane, and dichlomethane/methane (1 : 1) leaf and root extracts of* E. elephantina* for in vitro activity against* Plasmodium falciparum* using the parasite lactase dehydrogenase (pLDH) assay and chloroquine diphosphate (Sigma) as the positive control. The dichlomethane/methane (1 : 1) leaf and root extracts showed weak activity with IC_50_ values of 26 and 28 *μ*g/mL, respectively, while aqueous extracts for both leaves and roots showed weak activity with IC_50_ values >100 *μ*g/mL [62]. Although* E. elephantina* is widely used as traditional remedy for fever in Mozambique [25], South Africa [42], and Zimbabwe [43], the species did not display promising in vitro antiplasmodial activity, to support its traditional usage in the management and treatment of fever. A possible explanation could be that* E. elephantina* act as antipyretics or immune stimulants to relieve the symptoms of the disease, rather than having direct antiparasitic activity [73]. Alternatively, precursors of the active components may be present in* E. elephantina* extracts but have to be modified, usually in vivo, before activity is exhibited [63].

### 7.6. Antioxidant Properties

Mpofu et al. [54] evaluated antioxidant properties of* E. elephantina* using DPPH radical scavenging method with the yen and duh percentage inhibition values ranging from 33 to 72% for both methanol and aqueous extracts. This study carried out by Mpofu et al. [54] revealed that there were more extractable antioxidants using methanol compared to water as the solvent. The antioxidant activities demonstrated by* E. elephantina* rhizome extracts are probably due to the presence of flavonoids and phenolics [74]. Antioxidant properties displayed by* E. elephantina* could be due to the compound ethyl gallate** 4**. Ethyl gallate** 4** isolated from ethanol extract of* Acacia nilotica* Wild ex Del. subsp.* indica* (Benth.) Brenan leaves demonstrated antioxidant activities in several in vitro assays [75], revealing that the compound was a hydrogen donor, metal chelator, and free radical scavenger.

### 7.7. Antirickettsial and Antibabesial

Antibabesial and antirickettsial in vitro assay systems have been used to evaluate* E. elephantina* rhizome extracts. Naidoo et al. [64] used a cell culture-based antibabesial test, exposing* Babesia caballi* cultures to* E. elephantina,* and effectivity was established by the degree of inhibition using a colour change method as well as by evaluating percentage of parasitized cells on thin culture smears and calculating the degree of residual infectivity. The antibabesial drugs used as controls, imidocarb and diminazene, demonstrated efficacy, exhibiting EC_50_ values of 0.08 and 0.30 *μ*g/mL, respectively. Similarly,* E. elephantina* acetone rhizome extract demonstrated activity at 100 *μ*g/mL. Acetone rhizome extracts of* E. elephantina* demonstrated significant activity against a tick-borne disease that is problematic to the livestock of South African farmers [64].

Naidoo et al. [65] evaluated the antirickettsial activity of leaf acetone extracts of* E. elephantina* in an in vitro* Ehrlichia ruminantium* culture system.* Ehrlichia ruminantium* cultures were incubated with acetone extracts of the leaves and results were compared to those obtained with oxytetracycline and untreated controls.* Elephantorrhiza elephantina* possessed antiehrlichial activity with EC_50_ values of 111.40 *μ*g/mL and EC_90_ values of 228.90 *μ*g/mL. The EC_50_ and EC_90_ values for oxytetracycline were 0.29 and 0.08 *μ*g/mL. These results demonstrate that* E. elephantina* leaf extracts may be inhibitory against the* Ehrlichia* parasite by a similar mechanism to each other, which was unrelated to the mechanism of action of the tetracyclines [65].

### 7.8. Toxicity

Despite the long use of* E. elephantina* as herbal medicine in southern Africa to treat numerous human and animal diseases and ailments, the species is known to be harmful when used at an excessive dosage [3, 4, 48]. Root infusions of* E. elephantina* have been reported to have constipating effects [48] while seeds are strongly irritant and have been suspected of causing human death when used as herbal medicine [4]. According to Hutchings et al. [4] an aqueous extract of the seed equivalent to 0,75 g produced extensive necrosis at the point of injection and gastroenteritis and pulmonary oedema when injected subcutaneously in the guinea-pig. Symptoms of poisoning were apathy, loss of appetite, and profuse foetid diarrhoea with death occurring within twenty-four hours with the animal in a state of exhaustion. Postmortem examination revealed acute gastroenteritis with numerous haemorrhages and marked degeneration of the liver [4]. Jansen [16] reported that the seeds of* E. elephantina* are toxic to sheep with a lethal dose 250 g and rabbits (lethal dose 5–7.50 g/kg) causing gastroenteritis and pulmonary oedema.

Preliminary acute toxicity evaluation of root extract of* E. elephantina* using Wistar rats showed no physiological and behavioural changes in the animals and also no mortalities were recorded [62]. In another study, Maphosa et al. [76] evaluated the acute, subacute, and chronic toxicity of* E. elephantina* root extracts by oral route in male and female Wistar rats. The authors recorded no mortalities but changes in body weight and haematological and serum biochemical parameters between the control and treated animals were observed. In acute tests, Maphosa et al. [76] observed decreased respiratory rate at higher doses of 1600 mg/kg, and, in subacute tests, the root extract of* E. elephantina* caused an increase in white blood cells, monocytes, and serum levels of creatinine at higher doses of 400 and 800 mg/kg. In chronic toxicity,* E. elephantina* extracts caused increase in lymphocytes and platelets and changes were also noted in the body and organ weights in both subacute and chronic toxicities. Maphosa et al. [76] observed acute hepatitis, intracrystal deposition (reminiscent of oxalate crystals) with renal crystals and secondary ascending pyelonephritis in animals receiving 800 mg/kg in subacute toxicity tests while pulmonary granulomas were noted in animals which received 400 mg/kg. In chronic toxicity tests, Maphosa et al. [76] observed mild to moderate splenic siderosis, pulmonary granulomas, refractile crystal deposits, and associated ascending pyelonephritis. Mpofu et al. [54] evaluated cytotoxicity activity of* E. elephantina* using the brine shrimp lethality test. Chloroform rhizome extract of* E. elephantina* exhibited some degree of biological activity with LC_50_ value of 0.80 [54]. Based on toxicity evaluations done so far [54, 62, 76], it can be inferred that* E. elephantina* has some potential toxicity at certain dose levels and should be taken with caution when used as herbal medicine.

## 8. Conclusion

The present review summarizes the ethnomedicinal uses and recent findings on traditional uses, phytochemistry, pharmacology, and toxicity of different extracts and compounds of* E. elephantina*. Anthocyanidins, anthraquinones, esters, fatty acids, phenolic compounds, flavonoids, glycosides, polysterols, saponins, sugars, tannins, and triterpenoids have been demonstrated to be the main active ingredients of* E. elephantina*. Recent studies have focused on evaluating anthelmintic, antibacterial, antifungal, anti-inflammatory and antinociceptive, antiplasmodial, antioxidant, antibabesial, and antirickettsial activities of the different extracts and compounds isolated from the species. In the past 30 years,* E. elephantina* has been the subject of phytochemical and pharmacological research, and some of the traditional uses of this plant particularly against microbial infections and gastrointestinal parasites in animals have been validated by pharmacological studies. But there is not yet enough data on ethnopharmacological evaluation and clinical research on the species and few evaluations of target-organ toxicity have been documented. Most of the phytochemical and pharmacological evaluations have focused on rhizomes and roots of* E. elephantina*. The most important research gaps identified in this study are as follows:

1. Since* E. elephantina* is widely used in combination with other plant species in various herbal concoctions, there is need for extensive research to evaluate synergistic effects of the different extracts or pure isolates to evaluate their ability to enhance the efficiency of the additive mixtures,
2. Future research should also focus on aerial parts of the species in order to ensure full utilization of the possible medicinal potential of* E. elephantina*. There is need to investigate the chemical constituents and pharmacological effects of the bark, leaves, flowers, fruits, and seeds of* E. elephantina*.
3. Literature studies show that the major phytochemical compounds isolated from* E. elephantina* so far are mainly fatty acids, phenolic compounds, and esters, but very little attempt has been made to correlate the activities of these compounds with the ethnomedicinal uses of the species. Therefore, there is need for further research on different compounds isolated from* E. elephantina*; examples include fatty acids and esters. Detailed phytochemical studies of* E. elephantina* and its pharmacological properties especially the mechanism of action of its bioactive constituents to illustrate the correlation between its ethnomedicinal uses and pharmacological activities should be the focus of future research studies.
4. Extensive in vivo experiments are required to validate the existing pharmacological activities.
5. Since* E. elephantina* contain potentially toxic compounds, future studies should include the identification of toxic compounds, possible side effects caused by taking* E. elephantina* as herbal medicine, and mechanisms of how potential toxic components of the species can be managed.

## Figures and Tables

**Table 1:** Vernacular names of *Elephantorrhiza elephantina*.

Vernacular name(s), ethnic group or geographical region in brackets	Country	References
Elephant's foot (English), *chizezana, mosibe, mosidi, mositsane, mositsane tjizezana, motshijane* (Setswana)	Botswana	[21–23]
*Mositsane* (Sotho)	Lesotho	[24]
*Xivurayi* (Changana), dwarf elephant's root (English)	Mozambique	[25]
*Elandsboontjie* (Afrikaans), eland's bean (English), *gerbwürzel* (German), ||*an‡gâb* (Khoekhoegowab), *omundjoze* (Otjiherero)	Namibia	[26]
*Baswortel, elandsboontjie, leerbossie, looiersboontjie, olifantswortel* (Afrikaans), dwarf elephant's root, eland's bean, eland's wattle, elephant's foot, (English), *lešhitšana, mosehlana, mošitšana, motshitshane* (Sepedi), *gwejobomvu, mositsane* (Sotho, Tswana), *ntolwane* (Swazi), *intolwane, xixuvari* (Xhosa), *intolwane, intolwanu* (-enkulu), *ugweje, umdabu* (Zulu)	South Africa	[4, 8, 11, 17, 20, 27–33]
*Intolwane* (Swazi)	Swaziland	[34]
Elephant-root (English), *intolwane encinyane* (Ndebele), *chizezepasi, mupangara* (Shona)	Zimbabwe	[20, 35]

**Table 2:** Ethnomedicinal uses of *Elephantorrhiza elephantina* in southern Africa.

Use	Plant part(s) used and preparation	Country of practice	References
Abdominal pains	Rhizome, root decoction taken orally	Lesotho; Zimbabwe	[3, 24]
Acne	Rhizome, root infusion applied externally	South Africa	[15, 36–38]
Anemia	Root decoction taken orally	Mozambique	[25]
Aphrodisiac	Root decoction taken orally	Zimbabwe	[3]
Bleeding	Root decoction applied on affected body part	Lesotho	[39]
Bloody diarrhoea in children	Root powder wiped around anus	Botswana	[40, 41]
Blood pressure	Rhizome decoction taken orally	South Africa	[12]
Breast cancer	Rhizome decoction taken orally	Lesotho	[24]
Chest pains	Roots taken as emetics	South Africa	[42]
Cleans blood	Rhizome decoction taken orally	Lesotho	[24]
Cleaning the womb after abortion	Rhizome decoction taken orally	Botswana; South Africa	[36, 40, 41]
Clearing air canal	Rhizome decoction taken orally	South Africa	[12]
Constipation, heartburn, indigestion, loss of appetite, stomach ailments, vomiting	Ingredient of a herbal mixture known as “Sejeso” (Ingwe brand) which also includes *Alepidea amatymbica* Eckl. & Zeyh., *Hypoxis obtusa* Burch. ex Ker Gawl., *Pentanisia prunelloides* (Klotzsch & Eckl. & Zeyh.) Walp., deionized water and potassium sorbate as preservative	South Africa	[43]
Diarrhoea	Leaf, rhizome, root, stem decoction taken orally	Mozambique, South Africa, Swaziland	[15, 28, 29, 32, 34, 38, 44–46]
Diarrhoea	Rhizome mixed with root of *Acokanthera oblongifolia* Benth. & Hook.f. ex B.D. Jacks	South Africa	[44]
Dysentery	Root decoction taken orally	South Africa	[15, 38, 46]
Earache	Rhizome decoction taken orally	Botswana	[41]
Eczema	Roots and rhizome used in combination with *Pentanisia prunelloides* to treat eczema	South Africa	[36, 37]
Erectile dysfunction	Rhizome, root decoction taken orally	Botswana, South Africa	[21, 31, 47]
Fever	Roots taken as emetics	Mozambique, South Africa	[25, 42]
Fever	Rhizome decoction taken orally mixed with *Pentanisia prunelloides*	Zimbabwe	[43]
Haemorrhoids	Rhizome, root decoction taken orally	Lesotho, South Africa	[15, 24, 38, 48]
Herpes	Rhizome decoction taken orally	Lesotho	[24]
HIV/AIDS opportunistic diseases	Rhizome decoction taken orally mixed with roots of *Boscia albitrunca* (Burch.) Gilg & Gilg-Ben., *Peltophorum africanum *Sond. and *Plectranthus ciliatus *E. Mey.	South Africa	[49]
Itching	Rhizome decoction taken orally	South Africa	[12]
Infertility in women	Rhizome, root decoction taken orally	Lesotho, Zimbabwe	[3, 24]
Intestinal disorders	Rhizome, root decoction taken orally	Lesotho, South Africa	[15, 24, 38, 39]
Kidney failure	Rhizome decoction taken orally	South Africa	[12]
Love charms	Roots taken as emetics	South Africa	[42]
Menstrual problems	Root, stem decoction taken orally	Botswana, South Africa	[40, 44]
Pain killer	Root decoction taken orally	Mozambique	[25]
Peptic ulcers	Root decoction taken orally	South Africa	[4]
Rheumatic heart conditions	Root decoction taken orally	South Africa	[4]
Rheumatic heart conditions	Root decoction taken orally	South Africa	[4]
Sexually transmitted infections	Rhizome decoction taken orally	Botswana, Mozambique	[41, 45]
Shingles	Rhizome decoction taken orally	South Africa	[50]
Shingles	Root decoction taken orally in combination with *Cladostemon kirkii *(Oliv.) Pax & Gilg (roots),* Drimia delagoensis *(Baker) Jessop (bulb),* Sarcophyte sanguinea *Sparm. subsp.* piriei *(Hutch.) B. Hansen (bark)and *Ranunculus multifidus *Forssk. (whole plant)	South Africa	[51]
Sores	Rhizome decoction taken orally	South Africa	[50]
Sores	Root decoction taken orally in combination with *Cladostemon kirkii *(root),* Drimia delagoensis *(bulb), *Ficus sur *Forssk. (bark),* Ranunculus multifidus *(whole plant), *Sarcophyte sanguinea *subsp.* piriei *and *Senecio serratuloides *DC. (leaves)	South Africa	[51]
Stomach ailments	Roots taken as emetics	Lesotho, South Africa	[24, 42]
Stomach ailments	Rhizome decoction taken orally mixed with *Acokanthera oblongifolia* root or *Pentanisia prunelloides*	South Africa; Zimbabwe	[43, 44]
Sunburn	Underground parts used to treat sunburn	South Africa	[15, 38]
Syphilis	Root decoction taken orally	Lesotho, South Africa	[15, 24, 38, 39]
Tonsillitis	Rhizome boiled and extract taken orally	South Africa	[12]
Tuberculosis	Rhizome decoction taken orally	Lesotho	[24]
*Ethnoveterinary medicine*			
Appetite stimulant	Rhizome decoction	South Africa	[27]
Black quarter	Rhizome decoction	South Africa	[27, 30]
Cough	Rhizome decoction	South Africa	[52]
Diarrhoea	Rhizome decoction	South Africa	[30, 48, 52]
Dysentery in cattle and horses	Root decoction	South Africa	[48]
Ectoparasites in goats (mites, ticks)	Root decoction	South Africa	[33]
Gastrointestinal parasites	Rhizome decoction	South Africa	[30]
Gall sickness	Rhizome decoction	South Africa	[30]
Heartwater	Rhizome decoction	South Africa	[27, 52]
Mange	Root decoction given to cows	South Africa	[53]
Pneumonia	Rhizome decoction	South Africa	[52]
Retained placenta in cattle	Rhizome decoction	Botswana, South Africa	[22, 30]
Tonic	Rhizome decoction	South Africa	[27]

**Table 3:** Phytochemical compounds isolated from rhizomes or roots of *Elephantorrhiza elephantina*.

Phytochemical compounds	Extract	Method of compound characterization	References
*Anthraquinone*			
Anthraquinone **38**	Chloroform, methanol	LC-ESI-MS	[54]
*Ester*			
Ethyl gallate **4**	n-butanol	GC-MS	[41]
Butanedioic acid **19**	Hexane	GC-MS	[55]
Benzoic acid **20**	Hexane	GC-MS	[55]
3-phenyl-2-propenoic acid **21**	Hexane	GC-MS	[55]
Nonanedioic acid **22**	Hexane	GC-MS	[55]
Methyl 3-(3,5-di-tert-butyl-4-hydroxy-phenyl)propionate **27**	Hexane	GC-MS	[55]
*Fatty acid*			
Hexadecanoic acid **15**	Hexane	GC-MS	[55]
9,12-Octadecadienoic **16**	Hexane	GC-MS	[55]
9-Octadecenoic **17**	Hexane	GC-MS	[55]
Octadecanoic acid **18**	Hexane	GC-MS	[55]
Tridecanoic acid **23**	Hexane	GC-MS	[55]
Methyl pentadecanoate **24**	Hexane	GC-MS	[55]
Methyl hexadec-9-enoate **25**	Hexane	GC-MS	[55]
Methyl hexadecanoate **26**	Hexane	GC-MS	[55]
Cis-10-Heptadecenoic acid **28**	Hexane	GC-MS	[55]
Methyl heptadecanoate **29**	Hexane	GC-MS	[55]
Methyl octadecanoate **30**	Hexane	GC-MS	[55]
Cis-5,8,11,14,17-eicosapenta-enoic acid **31**	Hexane	GC-MS	[55]
Eicosanoic acid **32**	Hexane	GC-MS	[55]
Methyl tetracosanoate **33**	Hexane	GC-MS	[55]
Pentacosanoic acid **34**	Hexane	GC-MS	[55]
Hexacosanoic acid **35**	Hexane	GC-MS	[55]
Methyl octacosanoate **36**	Hexane	GC-MS	[55]
Tetradecanedioic acid **37**	Hexane	GC-MS	[55]
*Flavonoids*			
Dihydrokaempferol **1**	n-butanol	GC-MS	[41]
Kaempferol **2**	Ethanol, n-butanol	GC-MS, LC-ESI-MS	[41, 56]
*Glycoside*			
Ethyl-1-O-*β*-D-galactopyranoside 8	n-butanol	GC-MS	[41]
*Phenolic compounds*			
2-(3,4-Dihydroxyphenyl) ethanol **6**	n-butanol	GC-MS	[41]
Catechin **3**	Chloroform, methanol, n-butanol	GC-MS, NMR	[41, 57]
Gallic acid **5**	Chloroform, methanol, n-butanol	GC-MS, NMR	[41, 57]
4-Hydroxybenzoic acid **7**	n-butanol	GC-MS	[41]
Quercetin 3-O-*β*-D-glucopyranoside **9**	Chloroform, methanol, n-butanol	GC-MS, NMR	[41, 57]
Epigallocatechin gallate **44**	Ethanol	LC-ESI-MS	[56]
Quercetin **45**	Ethanol	LC-ESI-MS	[56]
Epicatechin gallate **46**	Ethanol	LC-ESI-MS	[56]
Methyl gallate **10**	Chloroform, methanol	NMR	[57]
3-O-Galloyl-3,3′,5,5′,7-pentahydroxyflavone **12**	Chloroform, methanol	NMR	[57]
Taxifolin-3′-O-*β*-D-glucoside **13**	Chloroform, methanol	NMR	[57]
Epicatechin **14**	Chloroform, ethanol, methanol	FTIR, LC-ESI-MS, NMR	[56, 57]
*Phytosterols*			
*β*-Sitosterol **11**	Chloroform, methanol	NMR	[57]
*Saponin*			
Diosgenin **40**	Chloroform, methanol	LC-ESI-MS	[54]
*Sugar*			
Rhamnose **41**	Chloroform, methanol	LC-ESI-MS	[54]
Glucuronic acid **42**	Chloroform, ethanol, methanol	LC-ESI-MS	[54, 56]
Arabinose **43**	Chloroform, ethanol, methanol	LC-ESI-MS	[54, 56]
*Triterpenoid*			
Oleanolic acid **39**	Chloroform, methanol	LC-ESI-MS	[54]
